# Elevated bilirubin levels are associated with a better renal prognosis and ameliorate kidney fibrosis

**DOI:** 10.1371/journal.pone.0172434

**Published:** 2017-02-22

**Authors:** Sehoon Park, Do Hyoung Kim, Jin Ho Hwang, Yong-Chul Kim, Jin Hyuk Kim, Chun Soo Lim, Yon Su Kim, Seung Hee Yang, Jung Pyo Lee

**Affiliations:** 1 Department of Biomedical Sciences, Seoul National University College of Medicine, Seoul, Korea; 2 Department of Internal Medicine, Seoul National University Seoul Metropolitan Government Boramae Medical Center, Seoul, Korea; 3 Department of Internal Medicine, Chung-Ang University Hospital, Seoul, Korea; 4 Kidney Research Institute, Seoul National University College of Medicine, Seoul, Korea; University Medical Center Utrecht, NETHERLANDS

## Abstract

**Background:**

Bilirubin has been reported to protect against kidney injury. However, further studies highlighting the beneficial effects of bilirubin on renal fibrosis and chronic renal function decline are necessary.

**Methods:**

We assessed a prospective cohort with a reference range of total bilirubin levels. The primary outcome was a 30% reduction in the estimated glomerular filtration rate (eGFR) from baseline, and the secondary outcome was a doubling of the serum creatinine levels, halving of the eGFR and the initiation of dialysis. In addition, experiments with tubular epithelial cells and C57BL/6 mice were performed to investigate the protective effects of bilirubin on kidney fibrosis.

**Results:**

As a result, 1,080 patients were included in the study cohort. The study group with relative hyperbilirubinemia (total bilirubin 0.8–1.2 mg/dL) showed a better prognosis in terms of the primary outcome (adjusted hazard ratio (HR) 0.33, 95% confidence interval (CI) 0.19–0.59, *P* < 0.001) and the secondary outcome (adjusted HR 0.20, 95% CI 0.05 to 0.71, *P* = 0.01) than that of the control group. Moreover, the bilirubin-treated mice showed less fibrosis in the unilateral ureteral obstruction (UUO) model (*P* < 0.05). In addition, bilirubin treatment decreased fibronectin expression in tubular epithelial cells in a dose-dependent manner (*P* < 0.05).

**Conclusions:**

Mildly elevated serum bilirubin levels were associated with better renal prognosis, and bilirubin treatment induced a beneficial effect on renal fibrosis. Therefore, bilirubin could be a potential therapeutic target to delay fibrosis-related kidney disease progression.

## Introduction

Chronic kidney disease (CKD) is currently a major morbidity in medicine. Prevention of CKD progression is crucial to avoid end-stage renal disease (ESRD), which severely impairs the patient’s quality of life and increases mortality [1]. Among the many mechanisms related to renal function decline, the development of fibrosis has been considered the major terminal pathway of CKD progression [2].

Many biomarkers have been described as related to CKD progression and renal fibrosis [3–5]. Among them, bilirubin induced a protective effect against kidney and cardiovascular disease [6]. The benefits of elevated serum bilirubin levels were reported in several clinical studies on patient prognosis [7–11] and were well-documented in patients with Gilbert syndrome [12–14]. However, few human studies have investigated the relationship between bilirubin levels and renal outcomes using a limited study group [9–11,15]. Moreover, there were conflicting results regarding the effects of bilirubin on the development of kidney fibrosis [16–18], although animal studies have suggested that bilirubin prevents fibrosis-related mechanisms [17,19,20]. Hence, additional studies with a generalized patient population that investigate the impacts of bilirubin on renal outcome are warranted to clarify the protective effects of bilirubin on renal outcomes.

In the current study, we aimed to reveal the effects of bilirubin on the progression of kidney dysfunction and renal fibrosis. We analyzed a prospective cohort with serum bilirubin levels within a reference range and assessed their renal outcomes. In addition, studies using C57BL/6 mice and human tubular epithelial cells (hTECs) were performed to determine whether kidney fibrosis is ameliorated by bilirubin treatment.

## Materials and methods

### Ethics statement

This study was approved by the Institutional Review Board of Seoul National University Boramae Medical Center (IRB No. 26-2015-113). The human study was conducted in accordance with the principles of the Declaration of Helsinki. As the clinical investigation was a prospective observational cohort study and did not include any medical interventions, informed consent was waived for medical record acquisition. All animal experiments were performed with the approval of the Institutional Animal Care and Use Committee of Seoul National University Boramae Hospital and were conducted in accordance with the Guidelines for the Care and Use of Laboratory Animals of the National Research Council.

### Study patients

We secondarily analyzed a prospective observational cohort of patients who visited our outpatient clinic for coronary vascular disease evaluation, including those who underwent coronary computed tomography (CT) angiography, from 2008 to 2013. The patients underwent a CT scan and other laboratory examinations, and their follow-up visit was scheduled by their attending physicians. Considering that our main aim was to assess long-term renal prognosis rather than cardiovascular outcomes or acute aggravation, we excluded the patients who had major cardiovascular events (MACE, defined by events of non-fatal myocardial infarction, cardiovascular death and stroke) during their follow-up periods. Patients with missing total serum bilirubin levels or with follow-up durations less than 1 month were not enrolled in the study. Among the several suggested reference ranges for the total bilirubin levels, we included patients with total serum bilirubin levels less than 1.3 mg/dL to exclude as many patients as possible with hidden causes of bilirubin elevation. Finally, patients with other diseases known to be related to elevated bilirubin levels, including liver cirrhosis, hepatitis virus infection, liver tumor, pancreatobiliary disease, and hemolytic disorders, were excluded. The patients with serum bilirubin levels greater than or equal to the median value (0.8 mg/dL) were defined as having mildly elevated serum bilirubin levels, and the others were included in the control group.

### Cohort data collection

At the time of the cohort enrollment, we collected the following demographic characteristics: age, sex, and baseline body mass index of the study group. Baseline histories of other medical comorbidities including hypertension, diabetes mellitus, coronary artery disease, stroke, liver cirrhosis, and cancer were documented. Use of medications at baseline was also recorded, including the use of angiotensin converting enzyme inhibitors (ACE I) or angiotensin receptor blocker (ARB), diuretic agents, and statins. Lastly, laboratory test results were collected at the time of coronary vascular evaluation including the following parameters: total serum bilirubin, calcium, albumin, aspartate aminotransferase (AST), alanine aminotransferase (ALT), alkaline phosphatase (ALP), total cholesterol, high density lipoprotein (HDL), low density lipoprotein (LDL), triglyceride (Tg), and serum creatinine (sCr). Estimated glomerular filtration rate (eGFR) values were calculated based on the measured creatinine levels using the Modification of Diet in Renal Disease (MDRD) equation [21].

### Clinical outcome assessment

The median follow-up duration of the study population was 3.9 (2.4–4.9) years. During the follow-up period, all measured sCr and calculated eGFR values were collected to assess renal outcomes compared to the baseline. All outcomes were defined to assess long-term renal prognosis; therefore, events that occurred 1 month after the coronary CT scan were evaluated, and the others were excluded from the study. The primary outcome was reduction of the eGFR by 30% from the baseline value. The secondary outcome was the occurrence of one of the following events: doubling of sCr levels, halving of the eGFR from baseline, and the initiation of dialysis. All outcomes including doubling of sCr and eGFR reduction were confirmed to be constant so as not to include transient changes. Patients with follow-up loss or death events were censored.

### Experimental animals and UUO model

Seven-week-old male C57BL/6 mice weighing 20 g were used. Five mice were included in each of the four groups according to whether the mice underwent unilateral ureteral obstruction (UUO) surgery and whether they received intraperitoneal bilirubin injections. 0.0584 g of unconjugated bilirubin (Sigma-Aldrich, St. Louis, MO, USA) was dissolved in 0.1 N NaOH and diluted with 10 mL PBS, which yielded a solution containing 2 mg/mL bilirubin at pH 8.0. A 30 mg/kg/day dose corresponding to a 300 μL volume was intraperitoneally injected 7 days before and after the sham or UUO surgery. The control group received vehicle injections. For the UUO surgery, the mice were first anesthetized with pentobarbital sodium (50 mg/kg, Nembutal; Abbott, Wiesbaden, Germany) and ketamine (100 mg/kg), and the surgery was performed by creating an incision in the left flank and ligating both the proximal and distal left ureter with a 4–0 silk suture. The mice in the sham operation group underwent the same surgical procedure, but the ureter was only isolated, not ligated. Twenty-four hours after the last bilirubin injection, the mice were euthanized by cervical dislocation, and blood samples and kidney tissues were collected. For histological analyses, 4-μm-thick paraffin sections of the C57BL/6 kidneys were stained with periodic acid-Schiff (PAS) and Masson’s trichrome (MT). The tubule-interstitial lesion index was scored by the standard point counting method. Additionally, an LMS510 META laser confocal microscope (Carl Zeiss, Jena, Germany) was used for the immunofluorescence study. The paraffin sections were stained with immunofluorescence antibodies against 4’,6-diamidino-2-phenylindole (DAPI), fibroblast-specific protein-1 (FSP1) and platelet-endothelial adhesion molecule-1 (CD31) (Molecular Probes, Eugene, OR, USA).

### hTECs

hTECs were isolated from tissues of resected kidneys from patients diagnosed with renal cell carcinoma. After dissecting the cortex, the unaffected specimens were minced and digested with Hank’s balanced salt solution (HBSS) containing 3 mg/mL collagenase (Sigma-Aldrich, St. Louis, MO, USA). After centrifugation for 5 minutes at 500 g, cortical tubular cells were isolated. The cells were then incubated in DMEM/F12. After 4 hours of incubation, the tubules were collected and cultured on collagen-coated petri dishes (BD Biosciences, Franklin Lakes, NJ, USA) until colonies of epithelial cells were established, and 2–3 passages were used in the current study. After starvation in serum-free media with 1% albumin for 24 hours, the hTECs were cultured for 48 hours in the presence of transforming growth factor beta (TGF-β, 2 ng/mL) in combination with bilirubin concentrations of 0, 0.85, and 1.7 μmol/L. Light microscopy images using a differential interference contrast optics camera (Leica, DFC-295) were acquired to observe morphologic changes in the hTECs. Immunofluorescence staining was then performed for DAPI, aquaporin 1 (AQP1), CD31, fibronectin, and collagen 1 (ABCAM, Cambridge, UK). The hTECs then underwent Western immunoblot analysis using primary antibodies targeting β-actin (Sigma-Aldrich, Saint Louis, MO, USA) and fibronectin (ABCAM, Cambridge, UK). For the assay, proteins were extracted, and equal amounts (80 μg) were separated on 10% sodium dodecyl sulfate (SDS)-polyacrylamide gels and transferred onto Immobilon-FL 0.4 μM polyvinylidene difluoride membranes (Millipore, Bedford, MA, USA). Anti-rabbit IgG (Vector Laboratories, Burlingame, CA, USA) secondary antibody was used. The amounts of fibronectin were normalized according to the amount of β-actin. Densitometric analysis was performed using the gel analysis process of ImageJ software (National Institutes of Health, Bethesda, Maryland, USA). We performed cell integrity assay, using the EarlyTox^™^ Cell integrity kit (Molecular Device, CA, USA) with live red dye for permeant cell membrane which marks both for live and dead cells and dead green dye for impermeant membrane and stains only cells with damaged outer membrane. The MetaMorph software version 7.8.10 (University Imaging Downingtown, PA, USA) was used to quantify the dead cells.

### Real-time PCR

Total RNA was extracted from both mouse kidney tissues and hTECs, and the mRNA levels were assessed by real-time PCR. Total RNA was isolated from the mouse kidneys or human tissues using the RNeasy kit (Qiagen GmBH, Hilden, Germany), and 1 μg of the RNA was reverse-transcribed. Using the ABI PRISM 7500 Sequence Detection System, real-time PCR was performed using Assay-on-Demand TaqMan probes. In mouse models, primers for TGF-β, FSP1, and glyceraldehyde-3-phosphate dehydrogenase (GAPDH) (Applied Biosystems, Foster City, CA, USA) were used. For in vitro studies, primers for fibronectin, Snail2 (ABCAM, Cambridge, UK), GADPH, Bax2, and Bcl-2 were used. The primer sequences for Bax2 and Bcl-2 are provided in the S1 Method. The mRNA expression level of each cytokine was normalized with respect to the GAPDH mRNA expression level.

### Statistical analysis

The data are presented as frequencies and percentages for categorical variables and were analyzed using chi-squared tests. Continuous variables were expressed as the mean (standard deviation) or median scores (interquartile ranges) depending on the results of the Shapiro-Wilk normality test and compared using Student's t-tests or the Mann-Whitney U tests according to their normality. A multivariable logistic regression test was performed for the variables that showed significant differences between the study and control groups. The relationship between the baseline total bilirubin levels and primary outcome risks was investigated using a penalized spline analysis [22]. Overall renal survival was assessed using a Kaplan-Meier survival curve with the log-rank method. For the multivariate analyses, the Cox regression proportional hazard method was used and adjusted for age, baseline serum creatinine levels and all variables that showed significant differences from the analysis of the baseline characteristics. Linear contrast analysis was performed to investigate the dose-dependent relationship of fibronectin protein amounts according to the bilirubin concentration. Statistical analyses were performed using R package version 3.2.5 (R Development Core Team, Austria). A two-sided *p*-value with a significance level of 0.05 was used.

## Results

### Study population

**Fig 1:**
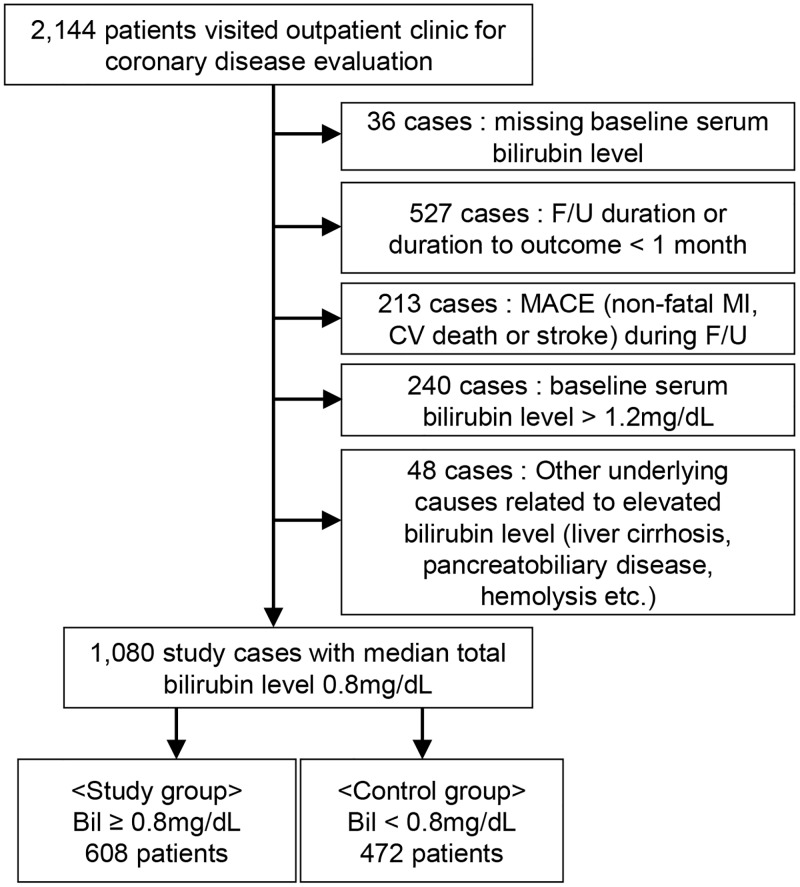
Flow diagram of the selected study population.

A diagram summarizing the study population selection is shown in Fig 1. A total of 2,144 patients visited outpatient clinics for coronary artery disease evaluation. Patients with a follow-up duration less than 1 month (*n* = 527) and patients who experienced MACE during the follow-up period (*n* = 213) were not enrolled in the study. After excluding patients with serum bilirubin levels higher than the reference range (*n* = 240), patients with other underlying diseases related to elevated bilirubin levels (*n* = 48), and patients for whom the bilirubin levels were not available (*n* = 36), a total of 1,080 patients with a median total bilirubin level of 0.8 mg/dL were included in the study cohort. Six hundred eight patients with serum bilirubin levels greater than or equal to the median value were included in the study group.

### Baseline characteristics

The baseline characteristics of our study population according to their serum bilirubin levels are shown in Table 1. The patients with relative hyperbilirubinemia were younger (*P* < 0.001), more frequently of the male sex (*P* < 0.001), and had higher levels of baseline laboratory parameters, including calcium (*P* < 0.001), albumin (*P* < 0.001), AST (*P* = 0.001), and ALT (*P* = 0.001), than those of the control group. There was no difference in the baseline levels of ALP, a sensitive marker for cholestasis, between the two groups (*P* = 0.90). Patients with mildly elevated bilirubin levels were less likely to have histories of hypertension (*P* = 0.001), diabetes mellitus (*P* < 0.001) and cancer (*P* = 0.006).

**Table 1:** Baseline characteristics of the study population.

	Bilirubin ≥ 0.8 (n = 629)	Bilirubin < 0.8 (n = 499)	*P*
Age (years)	61 (54–71)	66 (58–73)	< 0.001
Sex (male)	323 (53.1)	189 (40.0)	< 0.001
Body mass index (kg/m^2^)	24.8 (22.4–27.9)	24.4 (21.8–27.4)	0.18
Total bilirubin (mg/dL)	0.9 (0.8–1.1)	0.6 (0.5–0.7)	< 0.001
Serum creatinine (mg/dL)	0.90 (0.70–1.00)	0.80 (0.70–1.00)	0.10
eGFR (mL/min/1.73 m^2^)	84.5 (74.0–94.0)	82.0.3 (70.0–95.1)	0.10
Calcium (mg/dL)	9.0 (8.8–9.3)	8.9 (8.6–9.2)	< 0.001
Albumin (g/L)	4.2 (4.0–4.3)	4.1 (3.9–4.3)	< 0.001
AST (IU/L)	23 (20–29)	22 (18–27)	0.001
ALT (IU/L)	20 (15–28)	18 (14–26)	0.001
ALP (IU/L)	75 (63–91)	75 (63–93)	0.90
Total Cholesterol (mg/dL)	180 (149–206)	174 (139–202)	0.02
HDL (mg/dL)	44 (36–53)	43 (34–51)	0.08
LDL (mg/dL)	92 (52–133)	93 (57–131)	0.86
Tg (mg/dL)	96 (62–148)	97 (64–153)	0.47
Hypertension	516 (84.9)	433 (91.7)	0.001
Diabetes mellitus	51 (8.4)	74 (15.7)	< 0.001
Stroke	99 (1.5)	7 (1.5)	> 0.99
Coronary artery disease	33 (5.4)	26 (5.5)	> 0.99
Cancer	14 (2.3)	27 (5.7)	0.006
ACE I/ARBs	125 (20.6)	126 (26.7)	0.02
Diuretics	57 (9.4)	66 (14.0)	0.023
Statins	134 (22.0)	136 (28.8)	0.013

eGFR, estimated glomerular filtration rate, AST, aspartate aminotransferase, ALT, alanine aminotransferase, ALP, alkaline phosphatase, HDL, high density lipoprotein, LDL, low density lipoprotein, ACE I, angiotensin converting enzyme inhibitor, ARB, angiotensin receptor blocker

### Factors associated with serum bilirubin levels

Next, we determined which factors were associated with mildly elevated serum bilirubin levels (Table 2). The male sex (adjusted odds ratio (OR) 1.72, 95% confidence interval (CI) 1.29 to 2.28, *P* < 0.001) and an elevated baseline albumin level (adjusted OR 2.76, 95% CI 1.71 to 4.34, *P* < 0.001) were significantly associated with the presence of elevated bilirubin levels. In contrast, patients with histories of hypertension (adjusted OR 0.65, 95% CI 0.43 to 0.99, *P* = 0.04) and diabetes mellitus (adjusted OR 0.63, 95% CI 0.40 to 0.98, *P* = 0.04) did not tend to have elevated serum bilirubin levels.

**Table 2:** Clinical factors associated with mildly elevated serum bilirubin level.

	*OR	95% CI	P value
Age (years)	1.00	0.98–1.01	0.58
Sex (male)	1.72	1.29–2.28	< 0.001
Serum creatinine (mg/dL)	0.73	0.43–1.23	0.23
Calcium (mg/dL)	1.10	0.99–1.22	0.09
Albumin (g/L)	2.76	1.71–4.44	< 0.001
AST (IU/L)	1.01	0.99–1.01	0.50
ALT (IU/L)	1.00	0.99–1.01	0.55
Total cholesterol (mg/dL)	1.00	1.00–1.00	0.72
Hypertension	0.65	0.43–0.99	0.04
Diabetes mellitus	0.63	0.40–0.98	0.04
Cancer	0.61	0.30–1.22	0.16
ACE I/ARBs	0.95	0.67–1.36	0.79
Diuretics	1.19	0.76–1.85	0.45
Statins	0.94	0.67–1.32	0.74

OR, odds ratio, CI, confidence interval, AST, aspartate aminotransferase, ALT, alanine aminotransferase, ACE I, angiotensin converting enzyme inhibitor, ARB, angiotensin receptor blocker

*Adjusted with all variables in the table. All serum parameters were included in the analysis as continuous variables (natural units).

### Bilirubin levels and renal outcomes

**Fig 2:**
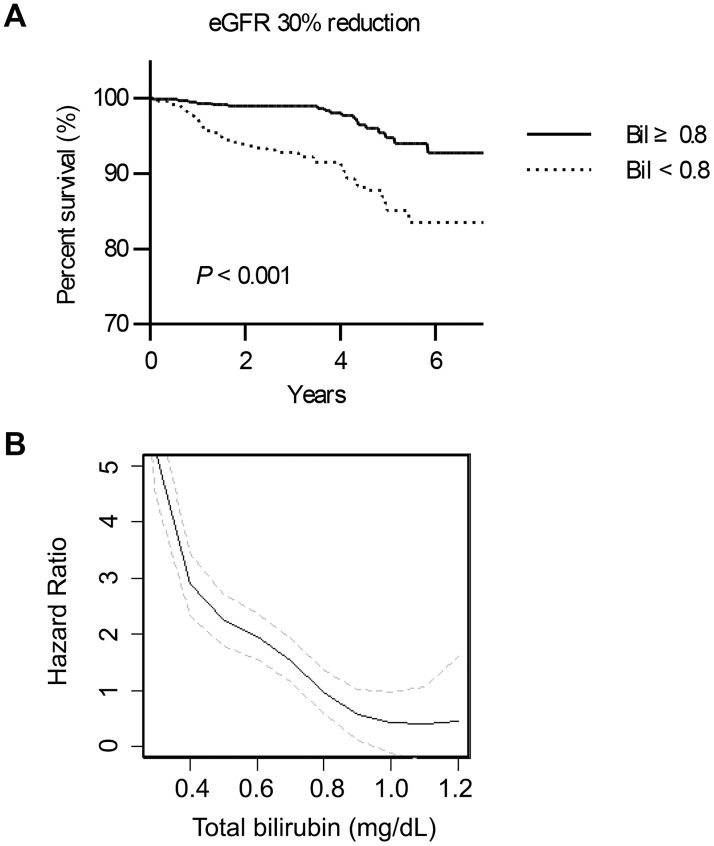
The renal outcome of the study patients. A) Kaplan-Meier survival curves of renal survival in terms of the primary outcomes. The x-axis shows the duration from the coronary CT angiography scan by years, and the y-axis shows the percent survival. B) Penalized spline models reveal the relationship between the baseline total serum bilirubin levels and the risk of primary outcomes. The linear line indicates the hazard ratio curve according to the bilirubin levels, and the gray broken lines above and below the linear line indicate the 95% confidence interval of the hazard ratio. The vertical dotted gray line indicates the serum bilirubin level of 0.8 mg/dL, which is the cut-off value for the study group in the current study.

During a median follow-up duration of 3.9 (2.4–4.9) years, there was a significant difference in renal survival according to whether the patients had mildly elevated bilirubin levels, both in terms of the primary outcome (*P* < 0.001, Fig 2A) and the secondary outcome (*P* = 0.02, data not shown in the figure). Furthermore, primary outcome risk was negatively correlated with serum bilirubin levels (Fig 2B). Namely, a higher serum bilirubin level was associated with a lower risk of a worse renal prognosis.

Next, as shown in Table 3, other well-known predictors were significantly associated with a worse primary outcome, including older age (HR 1.06, 95% CI 1.03–1.08, *P* < 0.001) and a history of diabetes mellitus (HR 2.48, 95% CI 1.29–4.77, *P* = 0.006). Higher serum albumin levels were associated with a lower risk of adverse renal outcomes (HR 0.18, 95% CI 0.11–0.29, *P* < 0.001). Interestingly, mildly elevated serum bilirubin levels were also a strong protective factor for renal prognosis in both the univariate (HR 0.29, 95% CI 0.17–0.50, *P* < 0.001) and multivariate (adjusted HR 0.33, 95% CI 0.19–0.59, *P* < 0.001) analyses. This relationship was the same for the secondary outcome (data not shown in the table, adjusted HR 0.20, 95% CI 0.05–0.71, *P* = 0.01). Again, when analyzed as a continuous variable, an increase of 1 mg/dL in serum bilirubin corresponded to protective effects in both primary (adjusted HR 0.12, 95% CI 0.04–0.38, P < 0.001) and secondary outcomes (adjusted HR 0.05, 95% CI 0.01–0.42, P = 0.006) in the study cohort. Lastly, we divided our study groups into three subgroups, the lower tertile (<0.7 mg/dL), middle tertile (0.7–0.9 mg/dL) and upper tertile (≥0.9 mg/dL) subgroups, and investigated whether the above linear relationship remained (S1 Table and S1 Fig). In agreement with the other analyses, the upper tertile subgroup (≥0.9 mg/dL) showed the best renal prognosis in terms of the primary outcome, and in fact, no secondary outcome was identified in the upper tertile subgroup. Considering the upper tertile group as the reference group, the primary outcome was significantly worse in the middle tertile subgroup (adjusted HR 2.77, 95% CI 1.26–6.10, *P* = 0.01). Interestingly, the prognosis was even worse in the lower tertile (adjusted HR 4.38, 95% CI 2.05–9.37, *P* < 0.001) subgroup, further suggesting the beneficial role of higher serum bilirubin levels.

**Table 3:** Clinical factors associated with primary outcome in the study cohort.

Variables	Univariable analysis			*Multivariable analysis		
	HR	95% CI	P value	HR	95% CI	P value
Age (years)	1.06	1.03–1.08	< 0.001	1.04	1.01–1.07	0.01
Sex (male)	1.34	0.83–2.17	0.24	1.83	1.07–3.11	0.03
Creatinine (mg/dL)	1.31	0.96–1.79	0.09	1.03	0.55–1.92	0.93
Hypertension	1.39	0.79–2.44	0.26	1.42	0.43–4.71	0.57
Diabetes mellitus	2.48	1.29–4.77	0.006	1.70	0.90–3.20	0.10
Albumin (g/L)	0.18	0.11–0.29	< 0.001	0.37	0.19–0.71	0.003
Bilirubin 0.8–1.2 mg/dL	0.29	0.17–0.50	< 0.001	0.33	0.19–0.59	< 0.001

HR, hazard ratio, CI, confidence interval, eGFR, estimated glomerular filtration rate

*Adjusted with age, sex, creatinine, calcium, albumin, AST, ALT, total cholesterol, baseline use of ACE I/ARBs, diuretics, statins, history of hypertension, diabetes mellitus, and cancer. All serum parameters were included in the analysis as continuous variables (natural unit).

### Effects of bilirubin treatment on kidney fibrosis in mice with UUO

**Fig 3:**
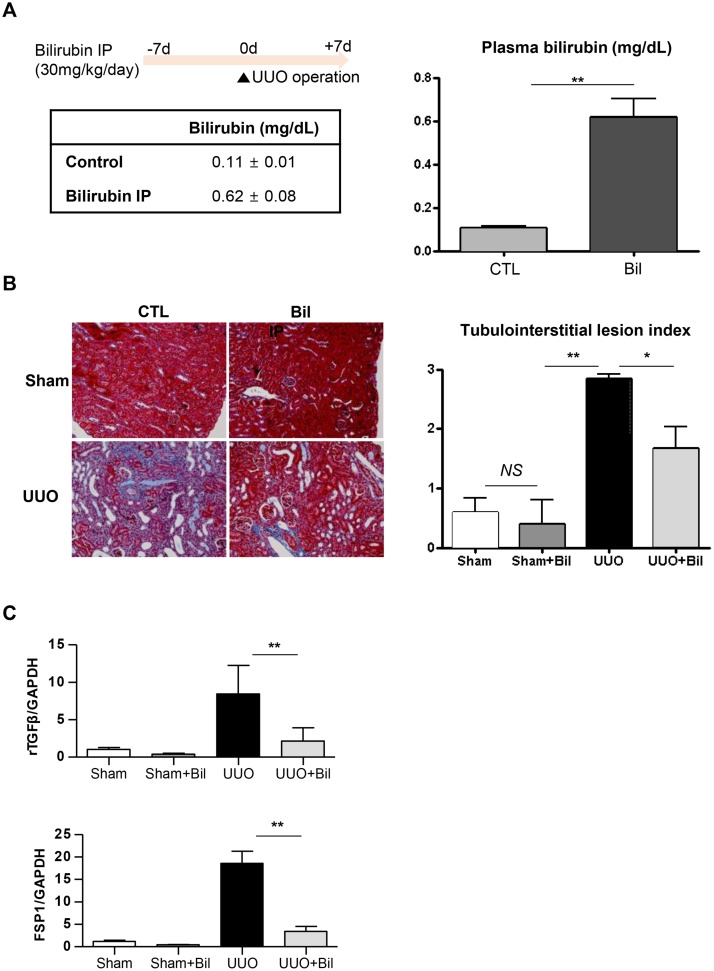
*In vivo* study in C57BL/6 mice. A) Study protocol and plasma bilirubin levels after intraperitoneal bilirubin administration. B) Representative images of histologic findings and the tubulointerstitial lesion index; left: the vehicle group, right: the bilirubin treated group, lower: a kidney after UUO surgery, upper: a contralateral (sham) kidney. C) Quantitative real-time PCR results for TGF-β and FSP1. CTL, control group; UUO, unilateral ureteral obstruction; Bil, bilirubin; hTECs, human tubular epithelial cells; NS, nonspecific; *P < 0.05, **P < 0.01. Each condition was evaluated in quadruplicate, and this figure represents one of four independent experiments.

Because renal fibrosis is one of the terminal endpoints related to kidney function decline [2], we further examined the relationship between the bilirubin levels and kidney fibrosis. After daily intraperitoneal bilirubin administration, there was a significant difference (*P* < 0.01) in plasma bilirubin levels between the treated group and the control group (Fig 3A). This disparity was also shown in histological findings after the UUO operation (Fig 3B). The UUO operation affected the tubule-interstitial lesion index of the mice *(P* < 0.01), and moreover, the index was significantly lower in the bilirubin-treated group than in the control group (*P* < 0.05). In immunofluorescence staining, although the UUO operation promoted fibroblast proliferation, the impact was markedly reduced in the bilirubin-treated group (S2 Fig). The above findings also correlated with the real-time PCR results (Fig 3C). The UUO surgery promoted the expression of TGF-β and FSP-1 (P < 0.005), implying that fibroblast proliferation increased after the surgery. However, the bilirubin treatment ameliorated this increase, and the treated group showed lower TGF-β (*P* < 0.01) and FSP-1 (*P* < 0.01) expression than the control group.

### Effects of bilirubin on hTEC fibrosis

**Fig 4:**
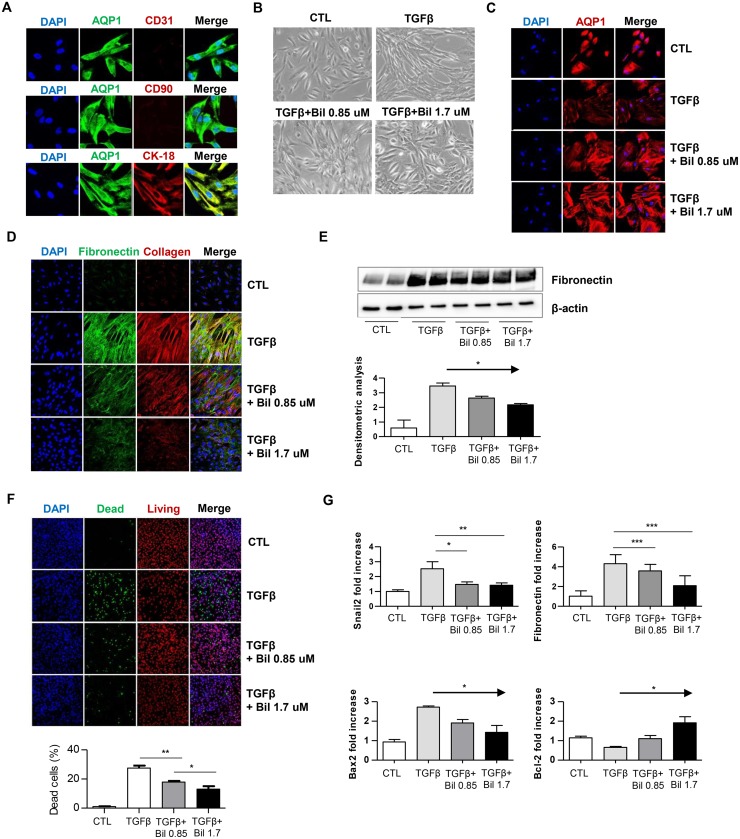
In vitro study with hTECs. A) Representative images of hTEC identification by immunofluorescence staining for CD31 as the endothelial cell marker, CD90 as the mesangial cell marker, and CK-18 as the proximal tubular cell marker. B) Representative differential interference contrast images of hTECs showing the morphological changes in hTECs cultured with rTGF-β in the presence or absence of bilirubin. Upper left picture: control group; upper right picture: hTECs cultured with rTGF-β; lower left picture: hTECs cultured with rTGF-β and 0.85 μmol/L bilirubin; lower right picture: hTECs cultured with rTGF-β and 1.7 μmol/L bilirubin. C) Representative immunofluorescence images of hTECs stained with AQP1. D) Representative immunofluorescence images of hTECs stained with DAPI, fibronectin and collagen 1. E) Western blot immunoassay for fibronectin and β-actin. F) Representative images of cell integrity assay. Live red dye for permeant cell membrane which marks both for live and dead cells, dead green dye for impermeant cell outer membrane and stains only cells with disrupted integrity G) Quantitative real-time PCR results for fibronectin, Snail2, Bcl-2, and Bax2. CTL, control, Bil, bilirubin.*P < 0.05, **P < 0.01, ***P < 0.005. Each condition was evaluated in triplicate, and this figure represents one of three independent experiments.

*In vitro* experiments were performed to further assess the effects of bilirubin on kidney fibrosis. Isolated hTECs were confirmed by identifying their phenotypes (Fig 4A), and then bilirubin was added to the culture medium of hTECs stimulated with TGF-β. TGF-β induced significant cytoskeletal remodeling and morphological changes in hTECs, and the cells became unstructured and elongated (Fig 4B). The TGF-β treatment also reduced the expression of AQP1, and the change recovered with bilirubin treatment (Fig 4C). Additionally, the TGF-β-treated cells produced substantial amounts of fibronectin and collagen 1 compared with those of the control group (Fig 4D). Interestingly, however, these changes were markedly decreased when the cells were cultured with additional bilirubin. Indeed, when quantified (Fig 4E), the amounts of fibronectin were decreased by bilirubin treatment in a dose-dependent manner (*P* < 0.05). In the cell integrity assay (Fig 4F), we observed the amount of dead cells were decreased with bilirubin treatment. As shown in the real-time PCR data (Fig 4G), fibronectin expression, as expected, was markedly increased when the hTECs were cultured with TGF-β, but the expression decreased in the presence of bilirubin. The effect was more prominent when the hTECs were cultured with 1.7 μmol/L bilirubin (*P* < 0.01). Furthermore, the expression of Snail2, a fibrosis-related transcription factor, was quantified, and the increased expression of Snail2 induced by TGF-β was normalized by bilirubin treatment (*P* < 0.005). The addition of bilirubin also ameliorated induced apoptosis represented by increased Bax2 expression and reduced Bcl-2 expression, by TGF- β in the hTECs, and the effect was more prominent when the hTECs were treated with 1.7 μmol/L bilirubin (P < 0.05).

## Discussion

In this study, we found that relative hyperbilirubinemia within a reference range was associated with delay of chronic renal function decline. Our experimental study supports these clinical data by demonstrating that bilirubin treatment ameliorates kidney fibrosis both in mice and in *in vitro* experiments. In addition, we found that bilirubin is a protective factor for renal disease progression, and the effect on renal fibrosis could be the underlying mechanism of this effect.

One strength of this study is that we showed the beneficial effect of serum bilirubin in an observational cohort comprising a large number of patients. The protective effect of the serum bilirubin levels on various fibrosis-related diseases has been studied and well-reviewed [6,8,12,13,23,24]. However, most of the studies considered cardiovascular disease [8,23,24], and few human studies have investigated kidney dysfunction progression according to serum bilirubin levels [10,11,15]. Although one previous study included a large number of patients with a median follow-up duration of 7 years [15], the study lacked several important clinical variables, such as the serum albumin level, which is significantly related to both proteinuria and bilirubin levels. As shown in the multivariable analysis of the results of the current study, mildly elevated total bilirubin levels were further confirmed to protect against kidney function decline.

There could be several reasons for the mildly elevated serum bilirubin levels. In the current study, male patients with higher serum albumin levels had relatively higher serum bilirubin levels. In contrast, histories of diabetes mellitus or hypertension were independently associated with lower serum bilirubin levels, and these relationships were also reported in previous studies [7,10,11].

Kidney fibrosis has been considered to be a common end-point of renal function decline or kidney aging [2,25]. Our experiments showed the protective effects of bilirubin on kidney fibrosis, which further supports our findings of the clinical study. Inflammation, hypertension, and oxidative injuries have been previously suggested as target mechanisms underlying the protective effects of bilirubin [13,16,17,19,20,26]. Additionally, bilirubin was related to wound healing in recent animal studies [27,28]. Nevertheless, the effects of bilirubin on renal fibrosis development vary according to study design [16–18]. By implementing a commonly used CKD model, UUO surgery [29], our results support the hypothesis that bilirubin is a potential therapeutic target to relieve fibrosis-related kidney injuries. Considering that our experiments was performed with unconjugated bilirubin and that previous major cohort studies were performed with patient with Gilbert’s syndrome [12,13], treatment with albumin-bound bilirubin might have potential benefits for renal fibrosis.

In our experiments, bilirubin decreased stimulated Snail2 expression in hTECs, and a significant alleviation of TGF-β-induced morphological changes in hTECs was observed. As Snail2 is a transcription factor with well-known roles in the mesenchymal-epithelial transition and fibrosis [25,30,31], bilirubin might affect fibroblast transformation. Moreover, another final outcome of cell dysregulation, apoptosis, induced by TGF- β was reduced by bilirubin treatment [32]. Considering that preventing fibrosis is regarded as a promising method to delay kidney disease progression, the effect of bilirubin on renal fibrosis might be the underlying mechanism of bilirubin’s protective effect on renal prognosis revealed in our clinical results. Also, as we analyzed patients with mild elevated total bilirubin levels and used low dose bilirubin treatments, only small degree of bilirubin elevation might be related to better outcomes in terms of kidney dysfunction, although exact beneficial dose range needs to be confirmed in further studies.

Our study has several limitations. First, we secondarily analyzed a prospective cohort composed of patients who initially visited the clinic for cardiovascular disease evaluation. Although we tried to control the confounding factors by excluding short-term events and patients with MACE during follow-up, performing the secondary analysis after a substantial number of patients were excluded might have affected our results. Nevertheless, by using this design, we excluded patients with other possible causes of bilirubin elevation to demonstrate the sole effect of bilirubin on renal outcome. Second, although bilirubin is a product of heme degradation, we did not include the hemoglobin level in our study because hemoglobin values were missing in many of the cohort records. Third, the follow-up protocol of the included patients was not systematized, as the study was an observational study, and therefore, possible delays in the outcome assessment may have been incurred for some patients. Further research with serial follow-ups would provide more valid information and reveal the threshold value of the beneficial effect of serum bilirubin. Fourth, the reference range of the total serum bilirubin levels varied between centers. However, when we also analyzed the cohort using the other commonly used criterion of 0.2–1.5 mg/dL, the result was similar (S3 Fig). In addition, the reason we excluded patients with serum bilirubin levels greater than 1.2 mg/dL was to evaluate the effect of mildly elevated serum bilirubin levels without hidden secondary causes. Lastly, there were unavoidable peritonitis induced by 2 weeks of bilirubin injection in our *in vivo* experiments, and this might have potentially affected the results. But, with appropriate use of solvent and pH adjustment, we observed only mild peritonitis at injection sites and no severe complications occurred. Also, better renal histopathologic finding despite the localized inflammation still supports our results.

In conclusion, mildly elevated serum bilirubin is a significant protective factor for renal prognosis. Additionally, in vivo and in vitro bilirubin treatment showed beneficial effects on kidney fibrosis, further highlighting the underlying mechanism of the beneficial effect of bilirubin. Therefore, bilirubin could be a potential therapeutic target to delay fibrosis-related kidney disease progression.

## Supporting information

S1 Fig The primary renal outcome according to the tertile bilirubin group. (TIF)

S2 Fig Immunofluorescence staining of C57BL/6 mice kidneys. (TIF)

S3 Fig The renal outcome of 1,477 patients with serum bilirubin 0.2–1.5 mg/dL. (TIF)

S1 File Provided data set of the study cohort. (CSV)

S1 Method The primer and probe sequences for Bax2 and Bcl-2. (DOCX)

S1 Table The association between tertile subgroups and the primary outcome. (DOCX)
