# Supplementary figures and images for: Elevated bilirubin levels are associated with a better renal prognosis and ameliorate kidney fibrosis
Source: PLoS One. 2017 Feb 22;12(2):e0172434. doi: 10.1371/journal.pone.0172434 (PMC5321406; doi:10.1371/journal.pone.0172434)

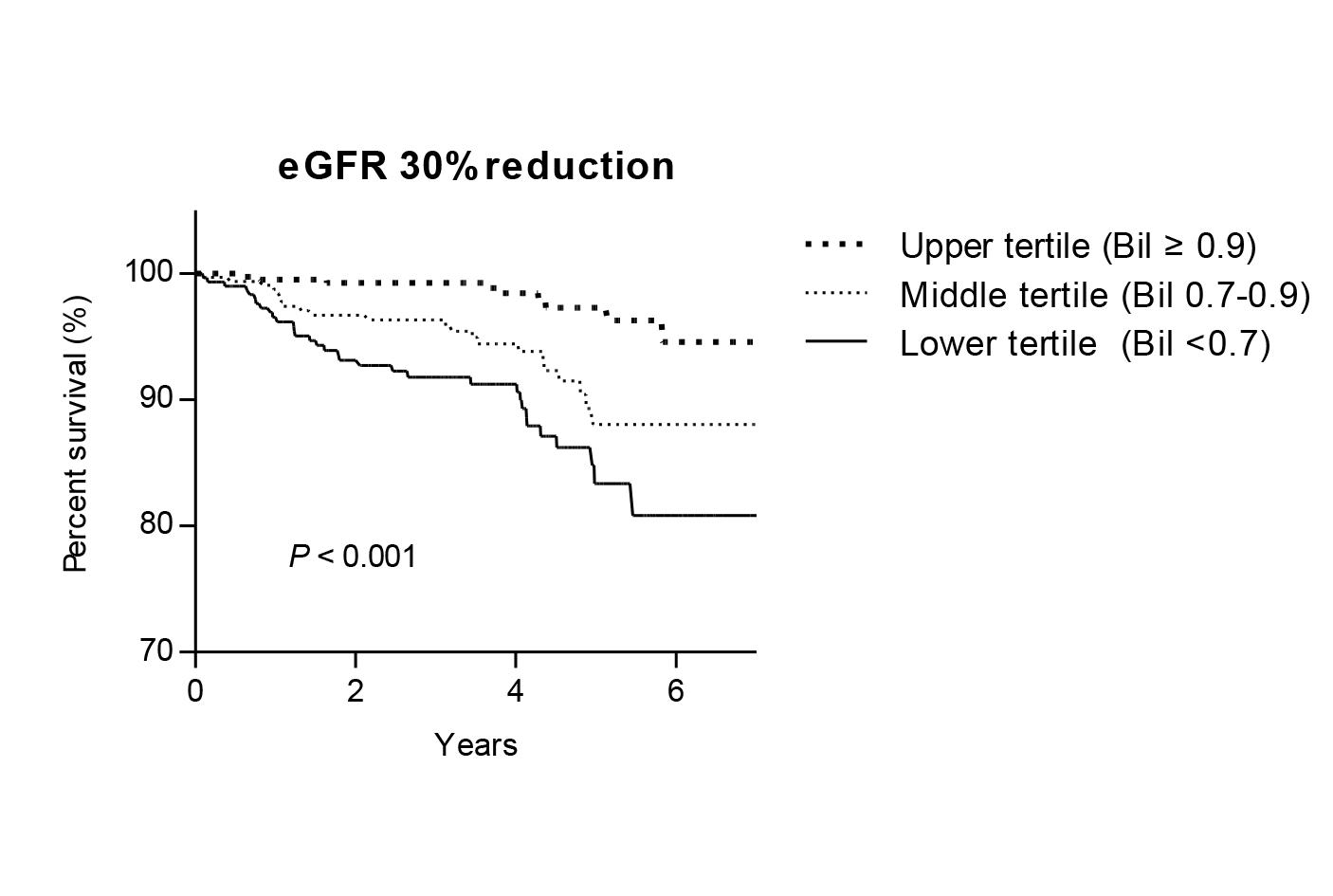

Supplement: S1 Fig — (TIF) [file pone.0172434.s001.tif]

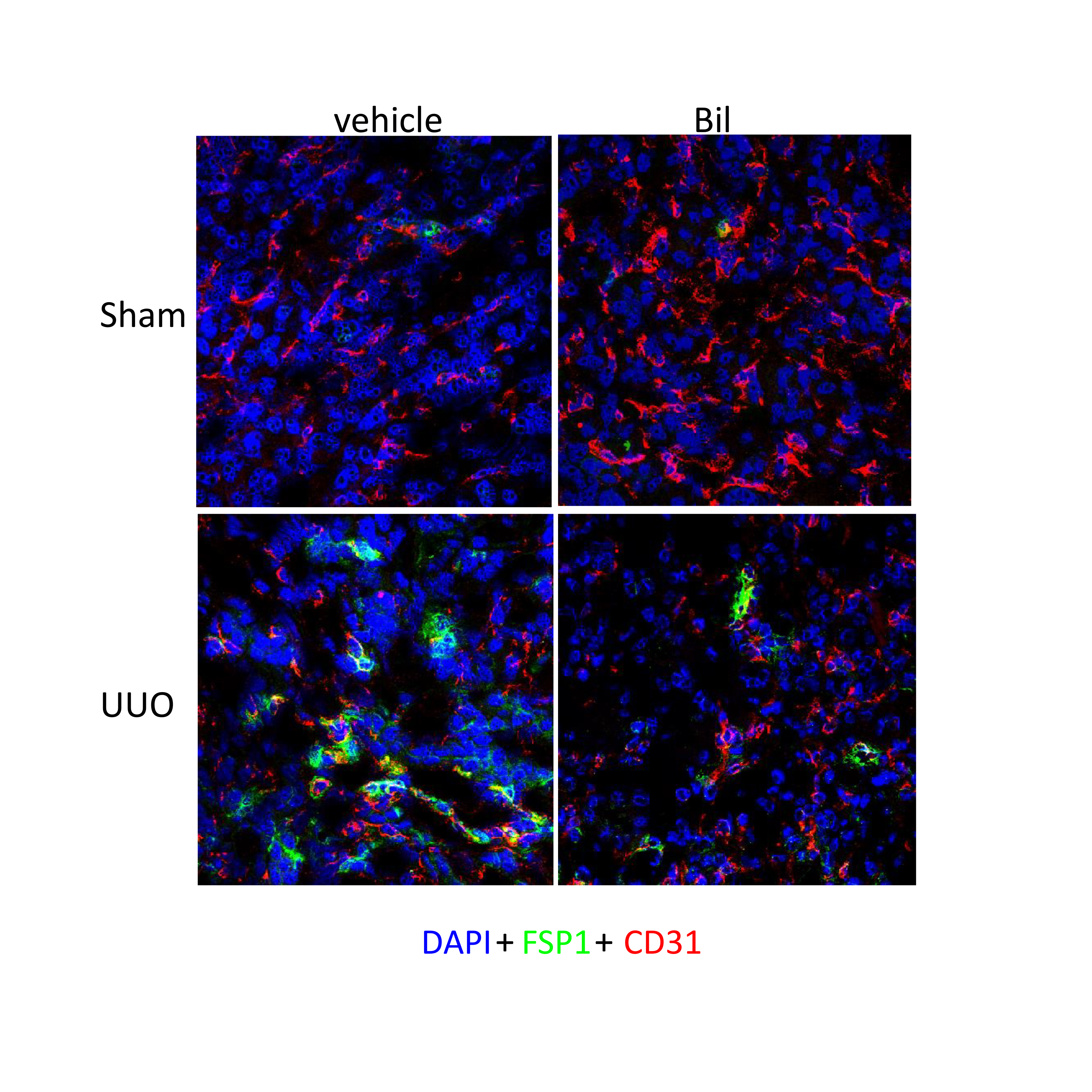

Supplement: S2 Fig — (TIF) [file pone.0172434.s002.tif]

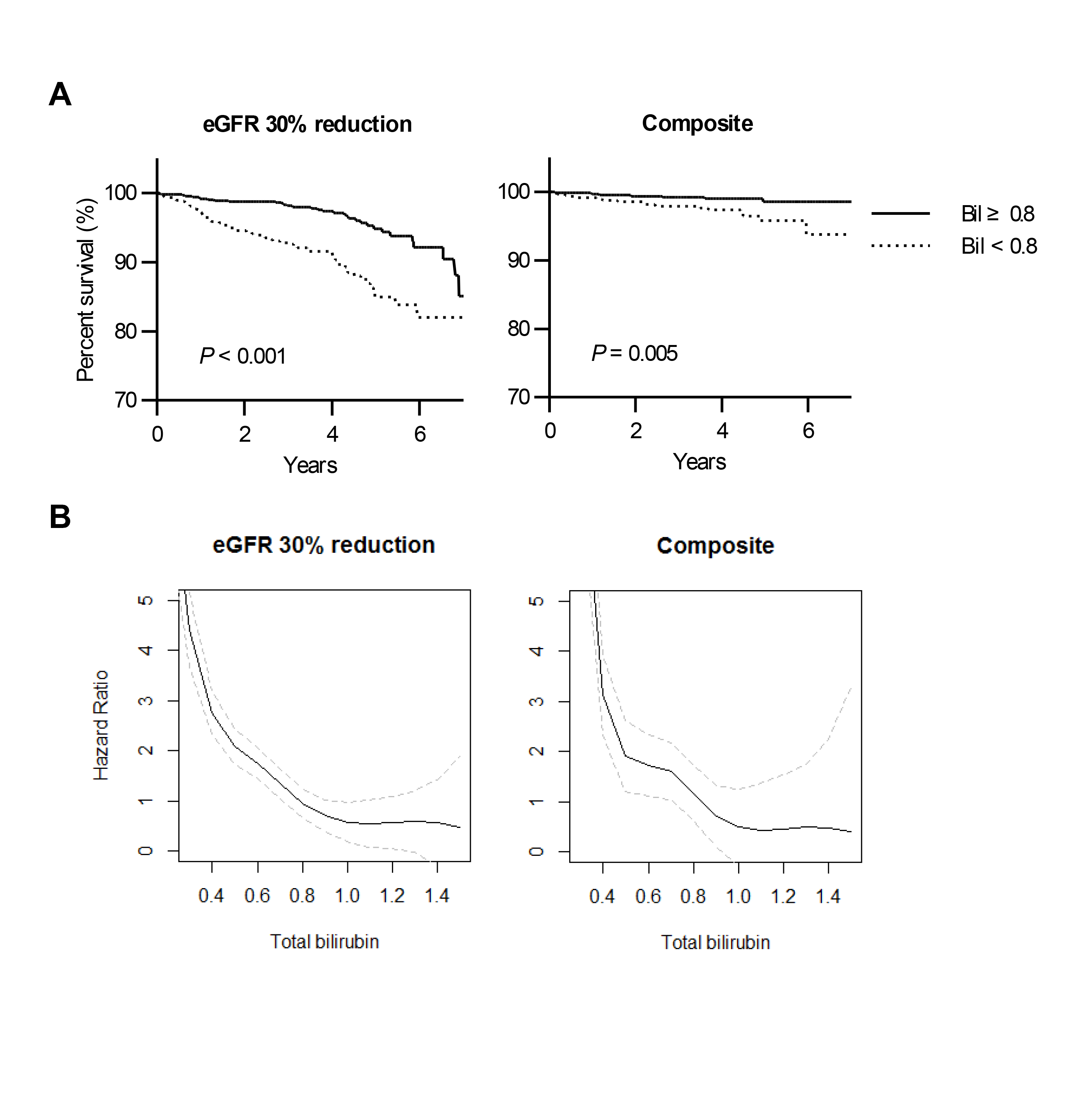

Supplement: S3 Fig — (TIF) [file pone.0172434.s003.tif]
