# Supplementary material for: Elevated bilirubin levels are associated with a better renal prognosis and ameliorate kidney fibrosis
Source: PLoS One. 2017 Feb 22;12(2):e0172434. doi: 10.1371/journal.pone.0172434 (PMC5321406; doi:10.1371/journal.pone.0172434)
Supplement: S1 Method — (DOCX) [file pone.0172434.s005.docx]

**S1 Method.** The primer sequence for Bax2 and Bcl-2.

Bax2

5-CCTGTGCACCAAGGTGCCGGAACT-3 (forward),

5-CCACCCTGGTCTTGGATCCAGCCC-3 (reverse),

5-TGGGCTGGACATTGGACTTCCTCCGGGAGCG-3 (probe)

Bcl-2

5- TTGTGGCCTTCTTTGAGTTCGGTG-3 (forward)

5- GGTGCCGGTTCAGGT ACTCAGTCA-3 (reverse),

5- TCGCCCCTGGTGGACAACATCGCCC-3 (probe)
