# Supplementary material for: Elevated bilirubin levels are associated with a better renal prognosis and ameliorate kidney fibrosis
Source: PLoS One. 2017 Feb 22;12(2):e0172434. doi: 10.1371/journal.pone.0172434 (PMC5321406; doi:10.1371/journal.pone.0172434)
Supplement: S1 Table — (DOCX) [file pone.0172434.s006.docx]

**S1 Table.** The association between the tertile subgroups and the primary outcome.

| Subgroups | Univariable analysis | | | ^*^Multivariable analysis | | |
| --- | --- | --- | --- | --- | --- | --- |
|  | HR | 95% CI | P value | HR | 95% CI | P value |
| Upper tertile (≥0.9 mg/dL) | Reference | | | Reference | | |
| Middle tertile (0.7-0.9 mg/dL) | 3.37 | 1.55-7.32 | 0.002 | 2.77 | 1.26-6.10 | 0.01 |
| Lower tertile (<0.7 mg/dL) | 5.79 | 2.77-12.10 | < 0.001 | 4.38 | 2.05-9.37 | < 0.001 |

HR, hazard ratio, CI, confidence interval

*Adjusted with age, sex, creatinine, calcium, albumin, AST, ALT, total cholesterol, baseline use of ACE I/ARBs, diuretics, statins, history of hypertension, diabetes mellitus, and cancer. All serum parameters were included in the analysis as continuous variables (natural unit).
